# Synthesis of New BINAP-Based Aminophosphines and Their ^31^P-NMR Spectroscopy

**DOI:** 10.3390/molecules18032788

**Published:** 2013-03-01

**Authors:** Christopher Anstiss, Peter Karuso, Mark Richardson, Fei Liu

**Affiliations:** Department Chemistry and Biomolecular Sciences, Macquarie University, Sydney, NSW, 2109, Australia

**Keywords:** bidentate ligands, BINAP aryl aminophosphines, substituent effects, NOBIN, MAP

## Abstract

BINAP aminophosphines are prevalent N,P-bidentate, chiral ligands for asymmetric catalysis. While modification via the BINAP-nitrogen linkage is well explored and has provided a diverse body of derivatives, modification of the other substituents of the phosphorous center is another avenue in generating new congeners of this important class of chiral ligands. Herein reported are new BINAP aryl aminophosphines with electron rich or deficient substituents on the aryl rings. This scalable synthesis converted readily available starting material, (*S*)-BINOL, to a key intermediate (*S*)-NOBIN, from which the final chiral aminophosphines were prepared via a palladium-catalyzed, phosphonylation reaction. The aryl substituents are able to modify the electronic properties of the phosphorous center as indicated by the range of ^31^P-NMR shifts of these new ligands. A computational analysis was performed to linearly quantitate contributions to the ^31^P-NMR shifts from both resonance and field effects of the substituents. This correlation may be useful for designing and preparing other related aminophosphines with varying ligand properties.

## 1. Introduction

Since the introduction of *C*_2_-symmetric phosphine ligands [1], much effort has been devoted to finding new chiral ligands for transition-metal catalysis [2,3,4]. Bidentate N,P-ligands are particularly effective and have been investigated extensively [5,6,7,8]. Among the variety of chiral backbones, BINAPs [2,2'-bis(diphenylphosphino)-1,1'-binaphthyls] are prominent scaffolds and considered “privileged” ligands [9,10]. The related BINAP-based, aminophosphine ligands (N,P bidentate instead of P,P-bidentate) emerged almost two decades ago and appeared in a wide range of asymmetric applications, such as allylic alkylation, cross-coupling, and hydrogenation reactions [11,12,13,14]. To expand the reaction scope of BINAP aminophosphines, new BINAP aminophosphines that can be readily diversified, as pioneered by the work of Kocovsky and co-workers in the synthesis of MAP (2-amino-2'-diphenylphosphino-1,1'-binaphthyl) [15], were also reported. Given the importance of MAP as a key entry into many chiral BINAP aminophosphines, others have reported its related syntheses [16,17]. BINAP aminophosphines in general have been used as ligands or nucleophilic phosphine catalysts [18,19,20,21,22,23,24].

Herein we report a synthesis of new congeners of the MAP class of BINAP aminophosphines **11b**, **11c**, and **11d**. The diphenyl substituents of MAP in this class are replaced with aryl substituents bearing electron rich or deficient groups. These phosphines exhibit a range of ^31^P-NMR chemical shifts. These new BINAP-based bidentate aminophosphines will lead to expanded applications in transition metal-complex formation and asymmetric catalysis.

## 2. Results and Discussion

### 2.1. Synthesis of Phosphine Oxides **1**–**3**

**Scheme 1:**
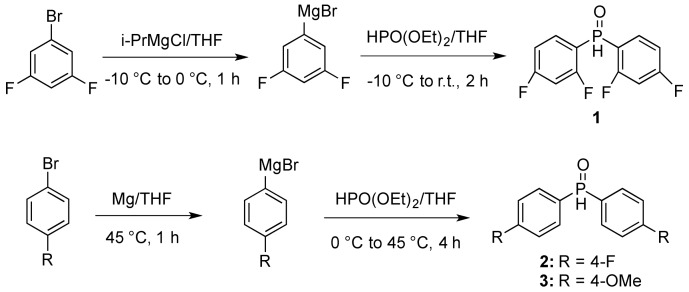
Synthesis of phosphine oxides **1**–**3**.

Secondary phosphine oxides are important precursors to phosphine ligands. They are either commercially available or readily prepared. The typical strategy for the synthesis of secondary phosphine oxides, such as **1**–**3**, is via substitution of diethyl phosphonate by alkyl or aryl Grignard reagents, as demonstrated by the groups of Busacca [25] and Hays [26] (Scheme 1). For the preparation of **3**, the procedure by Hays’ proceeded in 44% yield. Although Hays’ procedure was used here for the preparation of **2** and resulted in a low yield of 16%, Busacca and coworkers reported a gram-scale synthesis of **2** with 80% yield. The Busacca procedure was also used to prepare **1** in 83% yield at gram-scale.

### 2.2. Synthesis of Aminophosphines **11a**–**d**

**Scheme 2:**
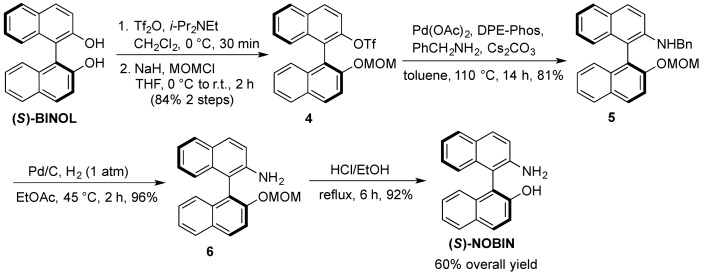
Synthesis of (*S*)-NOBIN from (*S*)-BINOL.

The bidentate, BINAP aminophosphine compounds **11a**–**d** were synthesized from the key intermediate (*S*)-NOBIN using conditions previously described by Kocovsky [27]. Whilst (*S*)-NOBIN is commercially available, it is relatively expensive (on average up to $250 per gram). Therefore a scalable synthesis of this key intermediate was performed from the much cheaper (S)-BINOL (approximately $1 per gram), using a modified procedure by Sälinger and Brückner [28] as we have previously reported (Scheme 2) [29]. It was found that the reduction of **5** at 10 mol% Pd on carbon for 2 h at 45 °C eliminated the over-reduction problem which occurred when the reduction was performed at 5% Pd on carbon for 7 h at 60 °C. This process consistently produced batches of (*S*)-NOBIN in good yields from (*S*)-BINOL at 10–20 mmol scale.

**Scheme 3:**
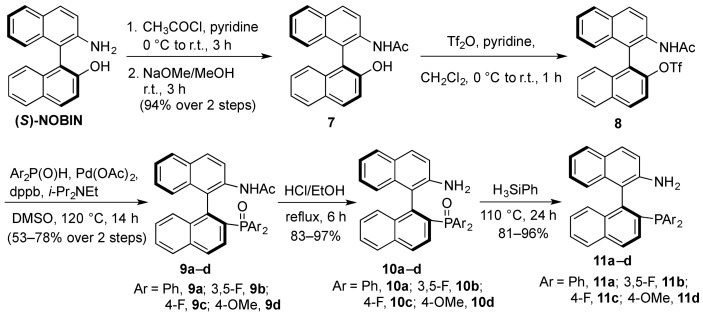
Synthesis of aminophosphines from (*S*)-NOBIN.

With (*S*)-NOBIN in hand, triflate **8** was synthesized in three steps as the key precursor to generating various aminophosphine derivatives via a palladium-catalyzed, phosphonylation reaction with the corresponding phosphine oxides (Scheme 3). The reaction with acetyl chloride proceeded with both *N*- and *O*-acetylation, and the undesired *O*-acetylation product was removed by mild hydrolysis to give **7** in excellent yield. From triflate **8**, the synthesis of *N*-acetylaminophosphine oxides **9a**–**d** proceeded smoothly in good yields (72–85%) under conditions reported by Kocovsky, with the exception of aminophosphine oxide **9b**. In this case, conditions reported by Maxwell and coworkers were required, which involve the coupling between bis(3,5-difluorophenyl)phosphine oxide and an aryl triflate using a 24-fold excess of Hünig’s base. Further, it was also found that an increased loading of palladium and dppb at 20–30 mol% or more was required for coupling to occur in moderate yield (54%). The *N*-acetylated phosphine oxides **9a**–**d** were converted by refluxing in ethanol/HCl into the free aminophosphine oxides **10a**–**d**, which were in turn converted to the corresponding aminophosphines **11a**–**d** via reduction in neat phenyl silane, which provided cleaner reduction products compared to those obtained by trichloro silane reduction. 

### 2.3. Phosphine NMR Spectroscopy and Computational Analysis of Aminophosphines **11a**–**d **

The ^31^P-NMR shifts were recorded for aminophosphines **11a**–**d ** (Table 1). In general, all aminophosphines displayed shifts upfield to that of the reference compound (H_3_PO_4_) with ^31^P chemical shifts ranging from −7.3 to −14.2 ppm. The substituent effects on the electronic properties of the phosphorous center have been investigated continuously since the work by Streuli and co-workers [30], who correlated the Brønsted basicity of phosphines to the sum of the Taft constant σ*. The difficulty of correlating ^31^P-NMR shifts with σ* has been recognized very early [31]. The σ* constant is essentially correlated to the inductive effect and it is clear from our work that the ^31^P chemical shift of **11c** is closer to **11d** than **11a**, suggesting that hyperconjugation (resonance effect) of the phenyl ring with the phosphorous 3d orbitals is more important than induction [32]. Kabachnik and co-workers developed a Taft constant (σ^ph^) specifically for organophosphorus compounds based on the ionization constants for about 150 phosphorus acids [33]. They reported that this constant gave a better fit for the p*K*_a_ and reactivity of phosphines than σ*. For compounds **11a**–**d**, we attempted to correlate the ^31^P chemical shift to the Taft constants σ* and σ*_I_* as well as eight other Hammett sigmas including σ^ph^, σ_m_, σ_p_, σ°, σ′, σ^+^, σ^−^, σ^•^ and the Taft steric parameter (E_S_), MR and π. The electronic parameters were found to dominate, as the aminophosphines considered here are arylphosphines with very similar steric parameters. The best correlation (R^2^ = 0.9418) was found with σ^−^ indicating that electronic factors, including the ability to stabilize a negative charge, are the most important factors in predicting the ^31^P chemical shift of simple triaryl phosphines. It should be noted that significant correlations were also found for (σ_p_ + σ_m_) and σ^+^ but no correlation was found with the other parameters. Clearly induction is of minor importance because the ^31^P chemical shift of **11c** (strongly electron withdrawing by induction) is closer to that of **11d** than was expected. Swain and Lupton [34] showed that these different sigma values are just linear combinations of resonance (R) and field/induction (F) parameters, which they calculated.

**Table 1:** ^31^P-NMR shifts and **σ** values ^a^ of the aminophosphines **11a**–**d**.

ID	^31^P δ (ppm)	σ*	σ*_I_*	σ^ph^	σ_m_	σ_p_	σ°	σ′	σ^+^	σ^−^	σ^•^	E_S_	MR	π
11a	−11.3	0	0.27	0	0	0	0	0.29	0	0	0	0	1.03	0
11b	−7.3	6.38	1.04	1.12	0.64	-	0.732	0.86	0.76	0.396	−0.22	−0.108	1.84	−0.276
11c	−13.2	3.19	0.52	0.56	-	0.06	0.48	0.43	−0.08	−0.03	−0.07	−0.55	0.92	0.14
11d	−14.2	1.77	0.27	−0.12	-	−0.27	−0.43	0.29	−0.78	−0.26	0.11	−0.55	3.98	−0.02

^a^ values taken from Hansch, C.; Leo, A; Hoekman, D. *Exploring QSAR: Hydrophobic, Electronic, and Steric Constants*. American Chemical Society: Washington DC, USA, 1995.

**Figure 1:**
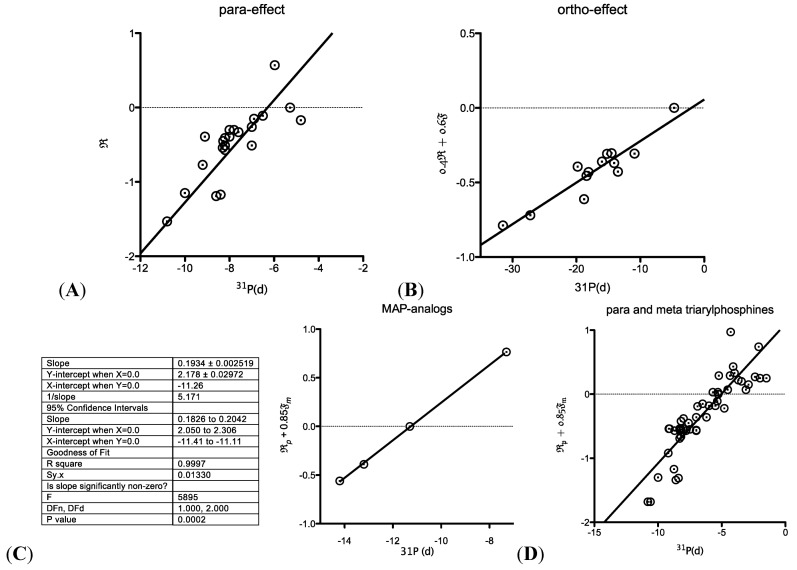
Correlation of ^31^P chemical shifts with Swain and Lupton Resonance (R) and Field (F) parameters. (**A**) correlation of para-substituted triarylphosphines with R; (**B**) of *ortho*-substituted triarylphosphines with 0.4R + 0.6F; (**C**) the best correlation between the ^31^P chemical shifts of **11a**–**d** was obtained against the Swain and Lupton parameters (R*_p_* + 0.85F*_m_*); (**D**) shows the correlation of *para*- and *meta*-substituted simple triarylphosphines against the same parameters.

Given that no simple method of predicting the chemical shift of phosphines has been reported we looked into this possibility a little more closely. Looking at the phosphorus chemical shifts reported by Maier *et al.* [35] we looked at their correlation to R and F. Surprisingly, for *para*-substituted triaryl phosphines (n = 23) the ^31^P chemical shift correlated with R, and *ortho-*substituted phosphines correlated best with 0.4F + 0.6 R (Figure 1A and B). No correlation was found with the *meta*-substituted triarylphosphines suggesting possibly other effects are at play here. Using these data, we looked at the correlation between R and F and the chemical shift of our *para*-substituted MAP analogs. A strongly linear correlation (R^2^ = 0.9998) was observed with R for the *para*-substituted MAP analogs (data not shown). Inclusion of any F component only made the correlation worse. Inclusion of **11b** with the Field parameter resulted in a linear regression (R^2^ = 0.9997) where the ^31^P chemical shift is proportional to R*_p_* + 0.85F*_m_*. Our data, albeit with a small dataset, suggests that the ^31^P chemical shifts of MAP analogs is related to the Swain and Lupton resonance and field parameters and that only the resonance parameter is important for *para*-substitution and only the field (induction) parameter is important in *meta*-substitution. To add weight to these finding, we used the ^31^P chemical shifts for *para*- and *meta*-substituted simple triarylphosphines [35] and plotted these against R*_p_* + 0.85F*_m_* and obtained a reasonable fit (R^2^ = 0.7473). This linear correlation, though totally empirical, may be useful when designing and preparing other congeners of this class of aminophosphines in the future with tunable electronic properties at the phosphine center.

## 3. Experimental

### 3.1. General

Linear regressions were carried out in GraphPad Prism v 5. (*S*)-BINOL, palladium acetate, palladium on carbon and dppb were purchased from Combi-Blocks, Inc. (San Diego, CA, USA). Diphenylphosphine oxide was purchased from Digital Specialty Chemicals (Toronto, Canada). Trifluoromethane sulfonic anhydride was purchased from Oakwood Products Inc. (West Columbia, Anaheim, CA, USA). 3,5-Difluorobromobenzene was purchased from AK Scientific. All other reagents were purchased from Sigma-Aldrich (Castle Hill, New South Wales, Australia). Unless specified, all commercially available reagents were used without further purification. Dichloromethane was distilled from calcium hydride. Tetrahydrofuran and toluene were distilled from sodium/benzophenone ketal. Diisopropylethylamine and pyridine were distilled from potassium hydroxide. Trifluoromethane-sulfonic anhydride was distilled from phosphorous pentoxide. All air and moisture sensitive reactions were performed under a nitrogen atmosphere. Reactions were magnetically stirred and monitored by thin-layer chromatography (TLC) using silica gel 60 F254 aluminium pre-coated plates (0.25 mm, Merck, Darmstadt, Germany). Flash column chromatography was performed on DAVISIL silica gel. All ^1^H, and ^13^C-NMR experiments were performed on a Bruker Avance DPX 400 MHz spectrometer. Chemical shifts were reported in ppm using CHCl_3_ (δ_H_; 7.26 ppm, (δ_C_; 77.5 ppm) as an internal reference. ^31^P-NMR spectroscopy was performed on a Bruker Avance DPX 400 MHz spectrometer at 298 K calibrated to H_3_PO_4_ (0 ppm). All spectra were processed using Bruker TOPSPIN (3). Infrared spectra were taken on a Thermo Fisher Scientific Inc. Nicolet iS10 smart iTR spectrometer and processed using OMNIC software, version 8.1. High resolution mass analysis was provided by University of Illinois, Urban-Champaign, IL, USA.

### 3.2. Preparations of Phosphinoxides **1–3**

*Bis(3,5-difluorophenyl)phosphine oxide* (**1**). Compound **1** was prepared as described by Busacca [25]. A flame dried 3-neck 250 mL round bottom flask equipped with a magnetic stirrer and 100 mL addition funnel was charged with aryl halide (17.3 mmol) and THF (10 mL) under N_2_ gas. The clear solution was cooled to −10 °C on an ice salt bath and the addition funnel was charged with *i*-PrMgCl (9.1 mL, 18.2 mmol) which was added to the cooled solution dropwise over a period of 8 min and then left to stir at 0 °C for 1 h. The reaction was re-cooled to −10 °C whereby the addition funnel was charged with diethylphosphite (675 μL, 5.24 mmol), and this reagent was added dropwise to the cooled solution over a period of 5 min, after which the reaction was allowed to warm to ambient temperature and stirred for 2 h. The reaction was cooled to 0 °C and quenched with careful addition of 3*N* HCl (10 mL). The mixture was extracted with methyl *tert*-butyl ether (3 × 10 mL) and the combined organic layers were dried (MgSO_4_) and concentrated. The oil was dried under high vacuum (0.01 mm Hg) overnight to yield the phosphine oxide as a white solid (1.2g, 83%) without further purification. ^1^H-NMR (400 MHz, CDCl_3_) δ 8.05 (d, *J* = 500 Hz, 1H), 7.07 (m, 2H), 7.23 (m, 2H), 7.27 (m, 2H); ^31^P-NMR (162 MHz, CDCl_3_) δ 18.8 (p, ^4^*J*_FP_ = 6.5 Hz).

*Bis(4-fluorophenyl)phosphine oxide* (**2**) and *bis(4-methoxy)phosphine oxide* (**3**) were prepared as described by Hays [26]. Magnesium turnings (85.1 mmol) in THF (20 mL) was stirred at 40 °C under argon for 1 h. To this Mg/THF slurry was added 4-bromoanisole (77.6 mmol) dropwise over 20 min. The mixture was then cooled to 0 °C and diethylphosphite (38.8 mmol) was added dropwise over 10 min and then stirred at 45 °C for 4 h. The reaction was carefully quenched with water and the resultant white mixture was diluted with ethyl acetate (EtOAc) and 10% HCl solution and stirred at room temperature for 30 min until the residual magnesium had disappeared. The product was extracted three times with EtOAc and the organic layer was washed with 2% HCl, brine and dried (MgSO_4_). Removal of solvent *in vacuo* afforded a pale yellow powder which was purified using flash chromatography (EtOAc/hexanes, 60% to 100%) to give the final phosphine oxide product.

*Bis(4-methoxy)phosphine oxide* (**3**). A chalky white solid, 4.50 g, 44% yield; ^1^H-NMR (400 MHz, CDCl_3_) δ 7.99 (d, *J =* 480 Hz, 1H), 7.57 (m, 4H), 6.95(d, *J* = 8.5 Hz, 4H), 3.81 (s, 6H); ^31^P-NMR (162 MHz, CDCl_3_) δ 23.1.

*Bis(4-fluorophenyl)phosphine oxide* (**2**). A yellow oil, 1.45 g, 16% yield; ^1^H-NMR (400 MHz, CDCl_3_) δ 8.08 (d, *J* = 480 Hz, 1H), 7.68 (m, 4H), 7.20 (m, 4H); ^31^P-NMR (162 MHz, CDCl_3_) δ 21.1.

### 3.3. Preparations of Aminophosphine Oxides **10a–d** and Aminophosphines **11a–d**

#### 3.3.1. Multigram Synthesis of (*S*)-NOBIN from (*S*)-BINOL

*(S)-2-Trifluoromethanesulfoxy-2′-methoxymethoxy-1,1′-binaphthyl* (**4**). To a solution of (*S*)-BINOL(5.02 g, 17.5 mmol) and diisopropylethylamine (3.05 mL, 17.5 mmol) in dichloromethane (90 mL) was slowly added triflic anhydride (2.94 mL, 17.5 mmol) at 0 °C under nitrogen. After stirring at room temperature for 1 h, the reaction was quenched with saturated aqueous sodium bicarbonate and extracted twice with dichloromethane. The combined organic extracts were washed with brine, dried over anhydrous magnesium sulfate and evaporated under reduced pressure. The crude triflate was dissolved in tetrahydrofuran (80 mL) under nitrogen and cooled to 0 °C whereby sodium hydride (505 mg, 21 mmol) was added portionwise. After stirring for 15 min at 0 °C chloromethylmethyl ether (1.60 mL, 21 mmol) was added dropwise and the mixture was left to stir at room temperature for 2 h. The reaction was carefully quenched with water and extracted three times with ethyl acetate. The combined organic extracts were washed with brine, dried over anhydrous magnesium sulfate and evaporated under reduced pressure. The crude product was purified by flash chromatography on silica gel, hexane:dichloromethane (2:1) to yield **4 **(6.84 g, 84%) as large white crystals. ^1^H-NMR (400 MHz, CDCl_3_) δ 3.23 (s, 3H), 5.04 (d, *J* = 7.0 Hz, 1H), 5.19 (d, *J* = 7.0 Hz, 1H), 7.05 (d, *J* = 8.5 Hz, 1H), 7.24 –7.29 (m, 1H), 7.33–7.40 (m, 3H), 7.52–7.57 (m, 1H), 7.58 (d, *J* = 9.0 Hz, 1H), 7.65 (d, *J* = 9.0 Hz, 1H), 7.87 (d, *J* = 8.0 Hz, 1H), 7.99 (d, *J =* 8.0 Hz, 1H), 8.03 (d, *J *= 9.5 Hz, 1H), 8.05 (d, *J* = 9.5 Hz, 1H).

*(S)-2-(N-Benzyl)amino-2′-methoxymethoxy-1,1′-binaphthyl* (**5**). A suspension of **4** (6.5 g, 14.1 mmol), palladium acetate (316 mg, 1.41 mmol), DPE-Phos (1.52 g, 2.82 mmol), cesium carbonate (5.52 g, 16.9 mmol) and benzylamine (1.85 mL, 16.9 mmol) in toluene (5 mL) was heated under nitrogen at 110 °C. After 14 h the reaction was cooled, diluted with toluene (30 mL), filtered through Celite and evaporated under reduced pressure. The crude product was purified by flash chromatography on silica gel, hexane:ethyl acetate (9:1) to yield **5 **(4.80 g, 81%) as a light yellow solid. ^1^H-NMR (400 MHz, CDCl_3_) δ 3.20 (s, 3H), 4.08 (t, *J* = 5.0 Hz, 1H), 4.42 (d, *J =* 5.0 Hz, 2H), 5.07 (dd, *J* = 21.5 Hz, 7.0 Hz, 2H), 6.97 (d, *J* = 8.0 Hz, 1H), 7.11–7.34 (m, 10H), 7.41 (t, *J* = 7.5 Hz, 1H), 7.63 (d, *J* = 9.0 Hz, 1H), 7.76 (d, *J* = 8.0 Hz, 1H), 7.81 (d, *J* = 9.0 Hz, 1H), 7.91 (d, *J *= 8.0 Hz, 1H), 7.99 (d, *J* = 9.0 Hz, 1H).

*(S)-2-Amino-2′-methoxymethoxy-1,1′-binaphthyl *(**6**). A suspension of **5 **(4.51 g, 10.8 mmol) and palladium acetate (10% on carbon, 1.10 g, 1.08 mmol) in ethyl acetate (25 mL) was heated under hydrogen gas (1 atm) at 45 °C for 3 h. After filtration through Celite, the solvent was evaporated under reduced pressure to afford **6 **(3.42 g, 96%) as a slightly yellow solid, which was used without further purification. ^1^H-NMR (400 MHz, CDCl_3_) δ 3.18 (s, 3H), 3.60 (bs, 2H), 5.05 (dd, *J* = 21.5 Hz, 7.0 Hz, 2H), 7.02 (d, *J* = 8.0 Hz, 1H), 7.10–7.31 (m, 5H), 7.39 (t, *J* = 7.5 Hz, 1H), 7.59 (d, *J* = 9.0 Hz, 1H), 7.75–7.84 (m, 2H), 7.90 (d, *J* = 8.0 Hz, 1H), 7.98 (d, *J* = 9.0 Hz, 1H).

*(S)-NOBIN. *To a solution of **6** (3.31 g, 10.1 mmol) in methanol (25 mL) and dichloromethane (25 mL) was added concentrated sulfuric acid (1.6 mL). After refluxing for 5 h, the reaction was cooled to room temperature, quenched with saturated aqueous sodium bicarbonate (pH 8–9) and extracted three times with dichloromethane. The combined organic extracts were washed with brine and dried over anhydrous magnesium sulfate and evaporated under reduced pressure to afford (*S*)-NOBIN (2.71 g, 94%) as a white solid which was used without further purification or recrystallized from benzene for spectroscopy. ^1^H-NMR (400 MHz, CDCl_3_) δ 3.70 (bs, 2H), 4.36 (bs, 1H), 7.06 (d, *J* = 8.0 Hz, 1H), 7.11 (d, *J* = 9.0 Hz, 1H), 7.16–7.42 (m, 6H), 7.81 (d, *J* = 7.5 Hz, 1H), 7.84 (d, *J* = 9.0 Hz, 1H), 7.89 (d, *J* = 8.0 Hz, 1H), 7.93 (d, *J* = 9.0 Hz, 1H).

#### 3.3.2. Synthesis of (*S*)-2-*N*-Acetyl-2′-hydroxy-1,1′-binaphthyl (**7**)

To a solution of (S)-NOBIN (4.94 g, 17.2 mmol) in pyridine (70 mL) was slowly added acetyl chloride (2.7 mL, 37.8 mmol) at 0 °C under nitrogen. After stirring at room temperature for 3 h, the reaction mixture was carefully poured onto ice cold water and extracted 3 times with dichloromethane. The extracts were washed twice with 5% aqueous hydrochloric acid, twice with water, twice with saturated aqueous sodium bicarbonate, brine, dried over anhydrous magnesium sulfate and evaporated under reduced pressure to afford the crude *N*,*O*-diacetate. To a solution of crude diacetate in methanol (450 mL) was added sodium methoxide (140 μL, 25% in methanol). After stirring at room temperature for 1 h, the solvent was evaporated under reduced pressure and the residue redissolved in dichloromethane. The organic layer was washed twice with water, dried over anhydrous magnesium sulfate and evaporated under reduced pressure to afford **7** (5.3 g, 94%) as an off-white solid that was used without further purification. ^1^H-NMR (400 MHz, CDCl_3_) δ 1.82 (s, 3H), 5.24 (bs, 1H), 6.91 (bs, 1H), 7.01 (d, *J* = 8.5 Hz, 1H), 7.14 (d, *J* = 8.5 Hz, 1H), 7.25–7.33 (m, 2H), 7.34–7.48 (m, 3H), 7.88–7.96 (m, 2H), 7.98 (d, *J* = 9.0 Hz, 1H), 8.04 (d, *J* = 9.0 Hz, 1H), 8.54 (d, *J* = 9.0 Hz, 1H); low resolution **ESI **[M+H^+^]: 328 (calcd. for (C_22_H_18_NO_2_)^+^, 328).

#### 3.3.3. Synthesis of Aminophosphine Oxides **10a–d**

To a solution of **7** (5.15 g, 15.7 mmol) and pyridine (3.82 mL, 47.1 mmol) in dichloromethane (80 mL) was slowly added triflic anhydride (2.91 mL, 17.3 mmol) at 0 °C under nitrogen. After stirring at room temperature for 3 h, the reaction was diluted with dichloromethane (80 mL). The organic layer was washed with 5% aqueous hydrochloric acid, saturated aqueous sodium bicarbonate, water, dried over anhydrous magnesium sulfate and evaporated under reduced pressure to afford the crude (*S*)-2-*N*-acetyl-2′-(trifluoromethanesulfonyloxy)1,1′-binaphthyl (**8**) which was used immediately for the phosphonylation reactions without further purification or characterization. To a suspension of the crude triflate (1.0 equiv.), phosphine oxide (2.1 equiv.), 1,4-bis(diphenylphosphino)butane (0.2 equiv.), palladium acetate (0.2 equiv.) in DMSO, 0.156 mmol/mL) was added diisopropylethylamine (3.7 equiv.). The reaction mixture was heated under nitrogen at 120 °C. After 12 h the reaction was cooled to room temperature, diluted with dichloromethane and the dark organic solution was washed twice with 5% aqueous hydrochloric acid, twice with water, saturated sodium bicarbonate, brine and dried (MgSO_4_). The filtrate was concentrated under reduced pressure and the residue was purified by flash chromatography on silica gel to afford the purified *N*-acetylphosphine oxide compounds **9a**–**d** which were used as intermediates to the aminophosphine compounds **10a**–**d**.

*(S)-2-N-Acetyl-2′-(diphenylphosphinoyl)-1,1′-binaphthyl* (**9a**). The crude triflate (4.30 g, 9.36 mmol), diphenylphosphine oxide (3.98 g, 19.7 mmol), dppb (798 mg, 1.87 mmol), palladium acetate (419 mg, 1.87 mmol) were suspended in DMSO (60 mL). Diisopropylethylamine (6.03 mL, 34.6 mmol) was added and the reaction heated at 120 °C. After 16 h, the reaction was worked up according to the general procedure and the crude residue was purified by flash chromatography on silica gel toluene/ethyl acetate (2:1 to 1:1) to yield **9a **(3.71 g, 78%) as an off white solid. ^1^H-NMR (400 MHz, CDCl_3_) δ 1.92 (s, 3H), 6.52 (d, *J*_1_ = 8.5 Hz, 1H), 6.60–6.67 (m, 2H), 6.77 (t, *J* = 7.5 Hz, 1H), 6.96 (t, *J* = 7.5 Hz, 1H), 7.11–7.25 (m, 5H), 7.41–7.75 (m, 8H), 7.86–8.00 (m, 4H); ^31^P-NMR (162 MHz, CDCl_3_) δ 28.9.

*(S)-2-N-Acetyl-2′-(bis(3,5-difluorophenyl)phosphinoyl)-1,1′-binaphthyl* (**9b**). The crude triflate (130 mg, 0.28 mmol), bis(3,5-difluorophenyl)phosphine oxide (115 mg, 0.42 mmol), 1,4-bis (diphenylphosphino)butane (36 mg, 0.08 mmol), palladium acetate (19 mg, 0.08 mmol) were suspended in DMSO (2.2 mL), to which was added diisopropylethylamine (1.17 mL, 6.72 mmol). The reaction mixture was heated under nitrogen at 120 °C. After 12 h the reaction was cooled to room temperature, diluted with dichloromethane and the dark organic solution was washed with saturated sodium bicarbonate, brine and dried (MgSO_4_). The filtrate was concentrated under reduced pressure and the residue was purified by flash chromatography on silica gel, ethyl acetate/hexanes (1:2 to 1:1) to afford **9b** (86 mg, 53%) as a beige solid. ^1^H-NMR (400 MHz, CDCl_3_) δ 1.89 (3H, s), 6.20 (t, *J* = 9.0 Hz, 1H), 6.55 (d, *J* = 8.5 Hz, 1H), 6.51–6.67 (m, 2H), 7.04–7.35 (m, 5H), 7.43–7.86 (m, 5H), 7.79 (m, 2H), 7.95 (d, *J* = 8.5 Hz, 1H), 8.03 (dd, *J* = 8.5 Hz, 2.0 Hz, 1H), 9.16 (bs, 1H); ^31^P-NMR (162 MHz, CDCl_3_) δ 27.3.

*(S)-2-N-Acetyl-2′-(bis(4-fluorophenyl)phosphinoyl)-1,1′-binaphthyl* (**9c**). The crude triflate (327 mg, 0.71 mmol), bis(4-fluorophenyl)phosphine oxide (350 mg, 1.49 mmol), dppb (61 mg, 0.14 mmol), palladium acetate (31 mg, 0.14 mmol) were suspended in DMSO (4.6 mL). Diisopropylethylamine (458 μL, 2.63 mmol) was added and the reaction heated at 120 °C. After 16 h, the reaction was worked up according to the general procedure and the crude residue was purified by flash chromatography on neutral alumina eluting with methanol/dichloromethane/toluene (1:4:15) to yield **9c **(283 mg, 73%) as a yellow solid. ^1^H-NMR (400 MHz, CDCl_3_) δ 1.91 (3H, s), 6.33 (t, *J* = 8.5 Hz, 2H), 6.51 (d, *J* = 8.5 Hz, 1H), 7.01 (t, *J* = 7.5 Hz, 1H), 7.08–7.28 (m, 6H), 7.44–7.77 (m, 6H), 7.89–7.99 (m, 4H), 9.49 (bs, 1H); ^31^P-NMR (162 MHz, CDCl_3_) δ 29.9.

*(S)-2-N-Acetyl-2′-(bis(4-methoxyphenyl)phosphinoyl)-1,1′-binaphthyl* (**9d**). The crude triflate (250 mg, 0.54 mmol), bis(4-methoxyphenyl)phosphine oxide (300 mg, 1.14 mmol), dppb (46 mg, 0.11 mmol), palladium acetate (24 mg, 0.11 mmol) were suspended in DMSO (3.5 mL). Diisopropylethylamine (351 μL, 2.01 mmol) was added and the reaction heated at 120 °C. After 16 h, the reaction was worked up according to the general procedure and the crude residue was purified by flash chromatography on silica gel toluene/ethyl acetate (1:2 to 1:99) to yield **9d **(225 mg, 73%) as a yellow solid. ^1^H-NMR (400 MHz, CDCl_3_) δ 1.92 (s, 3H), 3.54 (s, 3H), 3.88 (s, 3H), 6.13 (dd, *J* = 8.5 Hz, 2.0 Hz, 2H), 6.50 (d, *J* = 8.5 Hz, 1H), 6.96 (t, *J* = 7.5 Hz, 1H), 7.00–7.14 (m, 4H), 7.21 (t, *J* = 7.50 Hz, 2H), 7.48–7.59 (m, 3H), 7.65–7.77 (m, 2H), 7.81–7.96 (m, 4H), 9.77 (bs, 1H); ^31^P-NMR (162 MHz, CDCl_3_) δ 31.3.

To a solution of the appropriate *N*-acetylphosphine oxide **9a**–**d** in ethanol (0.1 mmol/mL) was added 5 M hydrochloric acid (0.025 mL of acid per mmol of phosphine oxide). After refluxing for 6 h, the reaction was cooled to room temperature, and quenched with saturated sodium bicarbonate to pH 9. The milky mixture was extracted three times with ether, and the combined organic layers were washed with brine and dried (MgSO_4_). The filtrate was evaporated under reduced pressure to afford the aminophosphine oxides **10a**–**d**.

*(S)-2-Amino-2′-(diphenylphosphinoyl)-1,1′-binaphthyl *(**10a**). A solution of phosphine oxide (700 mg, 1.37 mmol) in ethanol (14 mL) was added 5M hydrochloric acid (3.5 mL) was heated to reflux. After 6 h, the reaction was worked up according to the general procedure to afford aminophosphine **10a** (623 mg, 97%) as an off white solid. ^1^H-NMR (400 MHz, CDCl_3_) δ 3.88 (bs, 2H), 6.51 (d, *J* = 8.5 Hz, 1H), 6.76–6.82 (m, 2H), 6.87 (d, *J *= 8.8 Hz, 1H), 6.89–6.98 (m, 2H), 7.02 (t, *J* = 7.3 Hz, 1H), 7.20–7.30 (m, 4H), 7.35–7.50 (m, 5H), 7.55 (t, *J* = 7.3 Hz, 1H), 7.69–7.80 (m, 3H), 7.92 (d, *J* = 8.2 Hz, 1H), 7.96 (d, *J* = 8.8 Hz, 1H); ^31^P-NMR (162 MHz, CDCl_3_) δ 29.1.

*(S)-2-Amino-2′-(bis(3,5-difluorophenyl)phosphinoyl)-1,1′-binaphthyl* (**10b**). A solution of phosphine oxide (80 mg, 0.14 mmol) in ethanol (1.4 mL) was added 5M hydrochloric acid (350 μL) was heated to reflux. After 6 h, the reaction was worked up according to the general procedure to afford aminophosphine **10b** (66 mg, 89%) as a dull yellow solid. [**α**]**_D_^2^^0^** = +131 (*c* 1.0, CHCl_3_); ^1^H-NMR (400 MHz, CDCl_3_) δ 3.92 (bs, 2H), 6.37 (t, *J* = 8.7 Hz, 1H), 6.53 (d, *J* = 8.2 Hz, 1H), 6.73 (dd, *J* = 13.3, 5.3, 2H), 6.89–7.15 (m, 4H), 7.26–7.38 (m, 4H), 7.52 (d, *J* = 7.8 Hz, 1H), 7.48–7.76 (m, 3H), 7.98 (d, *J* = 8.3 Hz, 1H), 8.04 (dd, *J* = 8.8, 2.5 Hz, 1H); ^13^C-NMR (100 MHz, CDCl_3_) δ 106.7 (t, *J* = 25 Hz), 108.0 (t, *J* = 25 Hz), 113.4, 115.2, 118.9, 122.8, 124.5, 126.7, 127.5, 127.6, 128.1, 128.4, 128.6, 128.7, 129.2, 129.3, 129.5, 130.27, 131.1, 132.4, 133.5, 134.1, 135.1, 136.1, 137.3, 142.4, 145.0, 160.8, 161.8, 163.4, 164.4; ^31^P-NMR (162 MHz, CDCl_3_) δ 25.3 (p, *J* = 6.5 Hz). IR (cm^−^^1^) ν 1291, 3342; HRMS (ESI) [M+H^+^]: 542.1293, calcd for (C_32_H_21_NOPF_4_)^+^: 542.1297.

*(S)-2-Amino-2′-(bis(4-fluorophenyl)phosphinoyl)-1,1′-binaphthyl* (**10c**). A solution of phosphine oxide (359 mg, 0.66 mmol) in ethanol (6.7 mL) was added hydrochloric acid (5 M, 1.7 mL) was heated to reflux. After 6 h, the reaction was worked up according to the general procedure to afford aminophosphine **10c** (314 mg, 94%) as a light yellow solid. [**α**]**_D_^2^^0^** = +166 (*c* 1.0, CHCl_3_); ^1^H-NMR (400 MHz, CDCl_3_) δ 4.01 (bs, 2H), 6.45–6.58 (m, 3H), 6.94 (d, *J* = 8.7 Hz, 1H), 6.98 (t, 7.6 Hz, 1H), 7.02–7.14 (m, 3H), 7.18–7.34 (m, 4H), 7.44–7.54 (m, 2H), 7.58 (t, *J* = 7.6 Hz, 1H), 7.66–7.77 (m, 3H), 7.95 (d, *J* = 8.2 Hz, 1H), 8.00 (dd, *J* = 8.7 Hz, 2.5 Hz, 1H); ^13^C-NMR (100 MHz, CDCl_3_) δ 114.9, 115.9, 119.2, 122.6, 124.8, 126.6, 126.8, 127.4, 128.0, 128.1, 128.2, 128.6, 128.9, 129.1, 129.7, 130.5, 130.7, 131.8, 132.9, 133.5, 134.3, 134.5, 135.9, 141.4, 144.0, 163.4, 166.1; ^31^P-NMR (162 MHz, CDCl_3_) δ 28.2; IR (cm^−^^1^) ν 1158, 3324; HRMS (ESI) [M+H^+^]: 506.1478, calcd for (C_32_H_23_NOPF_2_)^+^: 506.1485.

*(S)-2-Amino-2′-(bis(4-methoxyphenyl)phosphinoyl)-1,1′-binaphthyl *(**10d**)*.* A solution of phosphine oxide (120 mg, 0.21 mmol) in ethanol (2.14 mL) was added hydrochloric acid (5 M, 536 μL) was heated to reflux. After 6 h, the reaction was worked up according to the general procedure to afford aminophosphine **10d** (91 mg, 83%) as a light yellow solid. [**α**]**_D_^2^^0^** = +174 (*c* 1.0, CHCl_3_); ^1^H-NMR (400 MHz, CDCl_3_) δ 3.65 (s, 3H), 3.84 (s, 3H), 3.94 (bs, 2H), 6.31 (dd, *J* = 8.7 Hz, 2.4 Hz, 2H), 6.52 (d, 8.7 Hz, 1H), 6.88 (dd, *J* = 8.7 Hz, 2.4 Hz, 2H), 6.91–7.00 (m, 2H), 7.06 (t, *J* = 7.5 Hz, 1H), 7.10–7.32 (m, 4H), 7.50 (t, *J* = 9.2 Hz, 2H), 7.55 (t, *J* = 7.5 Hz, 1H), 7.60-7.70 (m, 2H), 7.78 (dd, *J* = 11.2 Hz, 8.5 Hz, 1H), 7.93 (d, *J* = 8.5 Hz, 1H), 7.97 (dd, *J* = 8.5 Hz, 1.8 Hz, 1H); ^13^C-NMR (100 MHz, CDCl_3_) δ 113.3, 114.2, 119.43, 122.31, 122.7, 123.8, 124.3, 125.2, 126.3, 127.4, 127.8, 127.9, 128.3, 128.5, 128.7, 129.4, 129.6, 130.2, 132.0, 132.3, 132.4, 133.0, 133.5, 133.9, 134.0, 134.4, 135.7, 141.0, 143.8, 161.5, 162.3; ^31^P-NMR (162 MHz, CDCl_3_) δ 29.5; IR (cm^−^^1^) ν 1251, 3324; HRMS (ESI) [M+H^+^]: 530.1884, calcd for (C_34_H_29_NO_3_P)^+^: 530.1885.

#### 3.3.4. Synthesis of aminophosphines **11a**–**d**

The corresponding phosphine oxide **10a**–**d** was dissolved in neat phenyl silane and heated at 120 °C for 24 h. The dark reaction was dried down under a gentle stream of N_2_ gas to remove most of the phenyl silane, and the crude residue was purified by flash chromatography on silica gel to afford the purified aminophosphine compounds **11a**–**d**.

*(S)-2-N-Amino-2′-(diphenylphosphino)-1,1′-binaphthyl* (**11a**). The phosphine oxide **10a** (450 mg, 0.96 mmol) was heated in neat phenyl silane (900 μL) at 120 °C for 24 h. The residue was purified by flash chromatography on silica gel, dichloromethane/hexanes (1:2 to 1:1) to afford **11a** (418 mg, 96%) as a white solid. ^1^H-NMR (400 MHz, CDCl_3_) δ 3.29 (bs, 2H), 6.68 (d, *J* = 8.5 Hz, 1H), 6.96 (t, *J* = 7.5 Hz, 1H), 7.03 (d, *J* = 8.7 Hz, 1H), 7.05–7.21 (m, 7H), 7.24–7.36 (m, 6H), 7.45–7.53 (m, 2H), 7.74 (d, *J* = 8.0 Hz, 1H), 7.81 (d, *J* = 8.7 Hz, 1H), 7.90 (d, *J* = 8.5 Hz, 2H); ^31^P-NMR (162 MHz, CDCl_3_) δ −11.3.

*(S)-2-N-Amino-2′-(bis(3,5-difluorophenylphosphino)-1,1′-binaphthyl *(**11b**). The phosphine oxide **10b** (60 mg, 0.11 mmol) was heated in neat phenyl silane (150 μL) at 120 °C for 24 h. The residue was purified by flash chromatography on silica gel, dichloromethane/hexanes (1:2 to 1:1) to afford **11b** (42 mg, 72%) as a white solid. [**α**]**_D_^2^^0^** = +27 (*c* 1.1, CHCl_3_); ^1^H-NMR (400 MHz, CDCl_3_) δ 3.51 (bs, 2H), 6.49–6.62 (m, 3H), 6.67 (t, *J* = 8.7 Hz, 1H), 6.73–6.84 (m, 3H), 6.98 (t, *J* = 7.5 Hz, 1H), 7.10 (d, *J* = 8.7 Hz, 1H), 7.16 (t, *J* = 7.5 Hz, 1H), 7.31–7.46 (m, 3H), 7.58 (t, *J* = 7.5 Hz, 1H), 7.77 (d, *J* = 8.0 Hz, 1H), 7.85 (d, *J* = 8.8 Hz, 1H), 7.92–8.04 (m, 2H); ^13^C-NMR (100 MHz, CDCl_3_) δ 104.8 (m), 116.3 (m), 118.3, 122.7, 124.3, 126.7, 126.8, 127.8, 128.2, 128.4, 128.7, 129.6, 130.1, 130.4, 133.2, 134.6, 134.8, 134.9, 140.1 (m), 142.3, 142.9, 143.3, 162.0 (m), 164.5 (m); ^31^P-NMR (162 MHz, CDCl_3_) δ −7.3; IR (cm^−^^1^) ν 3379; HRMS (ESI) [M+H^+^]: 526.1342, calcd for (C_32_H_21_NF_4_P)^+^: 526.1348.

*(S)-2-N-Amino-2′-(bis(4-fluorophenylphosphino)-1,1′-binaphthyl *(**11c**). The phosphine oxide **10c** (115 mg, 0.23 mmol) was heated in neat phenyl silane (150 μL) at 120 °C for 24 h. The residue was purified by flash chromatography on silica gel, dichloromethane/hexanes (2:3 to 3:2) to afford **11b** (90 mg, 81%) as a white solid. [**α**]**_D_^2^^0^** = +22 (*c* 0.95, CHCl_3_); ^1^H-NMR (400 MHz, CDCl_3_) δ 3.25 (bs, 2H), 6.55 (d, *J* = 8.5 Hz, 1H), 6.78–6.89 (m, 2H), 6.93 (t, *J* = 7.5 Hz, 1H), 6.96–7.06 (m, 5H), 7.15 (t, *J* = 7.5 Hz, 1H), 7.20–7.37 (m, 4H), 7.41 (dd, *J* = 8.5, 2.8 Hz, 1H), 7.53 (t, *J* = 7.5 Hz, 1H), 7.74 (d, *J* = 8.5 Hz, 1H), 7.83 (d, *J* = 8.5 Hz, 1H), 7.93 (d, *J* = 8.5 Hz, 2H); ^13^C-NMR (100 MHz, CDCl_3_) δ 115.9 (m), 118.4, 122.6, 124.6, 125.6, 126.7, 127.5, 127.7, 128.3, 128.5, 129.1, 130.1, 132.6, 133.2, 133.7, 134.6, 135.9 (m), 137.3, 142.2, 162.3, 164.8; ^31^P-NMR (162 MHz, CDCl_3_) δ −13.2; IR (cm^−^^1^) ν 3379; HRMS **(**ESI**) **[M+H^+^]: 490.1528, calcd for (C_32_H_23_NF_2_P)^+^: 490.1536.

*(S)-2-N-Amino-2′-(bis(4-methoxyphenylphosphino)-1,1′-binaphthyl* (**11d**). The phosphine oxide **10d** (70 mg, 0.13 mmol) was heated in neat phenyl silane (100 μL) at 120 °C for 24 h. The residue was purified by flash chromatography on silica gel, 85% dichloromethane/hexane to afford **11d** (54 mg, 81%) as a white solid. [**α**]**_D_^2^^0^** = +31 (*c* 1.0, CHCl_3_); ^1^H-NMR (400 MHz, CDCl_3_) δ 3.22 (bs, 2H), 3.76 (s, 3H), 3.82 (s, 3H), 6.63 (d, *J* = 8.5 Hz, 1H), 6.70 (d, *J* = 8.5 Hz, 2H), 6.87 (d, *J* = 8.5 Hz, 2H), 6.91–7.03 (m, 3H), 7.06 (d, *J* = 8.5 Hz, 1H), 7.14 (t, *J* = 7.5 Hz, 1H), 7.18–7.35 (m, 4H), 7.43–7.55 (m, 2H), 7.75 (d, *J* = 8.5 Hz, 1H), 7.81 (d, *J* = 8.5 Hz, 1H), 7.91 (d, *J* = 8.5 Hz, 2H); ^13^C-NMR (100 MHz, CDCl_3_) δ 55.6, 55.7, 114.4, 118.6, 122.5, 124.9, 126.5, 126.6, 127.3, 127.4, 128.2, 128.3, 128.5, 128.7, 129.1, 129.2, 129.9, 130.4, 133.3, 134.4, 134.6, 135.6, 138.5, 141.0, 141.4, 142.4, 160.3, 160.4; ^31^P-NMR (162 MHz, CDCl_3_) δ −14.2; IR (cm^−^^1^) ν 3374; HRMS (ESI) [M+H^+^]: 512.1774, calcd for (C_34_H_27_NO_2_P)^+^: 512.1779.

## 4. Conclusions

We report here syntheses of new bidentate BINAP aminophosphines derived from (*S*)-NOBIN. These will add as congeners to a very important class of MAP-type compounds and lead to different aminophosphine derivatives as transition metal ligands or catalysts. The ^31^P shifts of these phosphines vary due primarily to resonance effects, but also some field (inductive) effects become important in the *meta* position, and a linear correlation has been extrapolated with appropriate coefficients to describe the contribution from either effect. This correlation does not correspond to any common Hammett constant (σ) and may be useful in designing new congeners of this class of MAP compounds with desired electron density on phosphorus.
